# Quantitative image-based collagen structural features predict the reversibility of hepatitis C virus-induced liver fibrosis post antiviral therapies

**DOI:** 10.1038/s41598-023-33567-4

**Published:** 2023-04-19

**Authors:** Laurent Gole, Feng Liu, Kok Haur Ong, Longjie Li, Hao Han, David Young, Gabriel Pik Liang Marini, Aileen Wee, Jingmin Zhao, Huiying Rao, Weimiao Yu, Lai Wei

**Affiliations:** 1 Institute of Molecular and Cell Biology, A*STAR, 61 Biopolis Drive, Proteos Building, Singapore, 138673 Singapore; 2 Peking University People’s Hospital, Peking University Hepatology Institute, Beijing Key Laboratory of Hepatitis C and Immunotherapy for Liver Diseases, No. 11, Xi Zhimen South Street, Beijing, 100044 People’s Republic of China; 3 Department of Pathology, Yong Loo Lin School of Medicine, National University of Singapore, National University Hospital, Singapore, Singapore; 4 Department of Pathology, The Fifth Medical Center of PLA General Hospital, Beijing, 100039 China; 5 Department of Hepatobiliary and Pancreatic Center, Beijing Tsinghua Changgung Hospital, School of Clinical Medicine, Tsinghua University, Beijing, 102218 China; 6 Bioinformatics Institute, A*STAR, Singapore, Singapore

**Keywords:** Inflammatory diseases, Computational models, Data acquisition, Image processing, Computational biology and bioinformatics, Translational research, Liver cirrhosis, Liver fibrosis, Hepatitis, Autoimmune hepatitis, Viral hepatitis

## Abstract

The novel targeted therapeutics for hepatitis C virus (HCV) in last decade solved most of the clinical needs for this disease. However, despite antiviral therapies resulting in sustained virologic response (SVR), a challenge remains where the stage of liver fibrosis in some patients remains unchanged or even worsens, with a higher risk of cirrhosis, known as the irreversible group. In this study, we provided novel tissue level collagen structural insight into early prediction of irreversible cases via image based computational analysis with a paired data cohort (of pre- and post-SVR) following direct-acting-antiviral (DAA)-based treatment. Two Photon Excitation and Second Harmonic Generation microscopy was used to image paired biopsies from 57 HCV patients and a fully automated digital collagen profiling platform was developed. In total, 41 digital image-based features were profiled where four key features were discovered to be strongly associated with fibrosis reversibility. The data was validated for prognostic value by prototyping predictive models based on two selected features: Collagen Area Ratio and Collagen Fiber Straightness. We concluded that collagen aggregation pattern and collagen thickness are strong indicators of liver fibrosis reversibility. These findings provide the potential implications of collagen structural features from DAA-based treatment and paves the way for a more comprehensive early prediction of reversibility using pre-SVR biopsy samples to enhance timely medical interventions and therapeutic strategies. Our findings on DAA-based treatment further contribute to the understanding of underline governing mechanism and knowledge base of structural morphology in which the future non-invasive prediction solution can be built upon.

## Introduction

According to the World Health Organization, about 58 million patients are infected with hepatitis C virus (HCV) globally, with an estimated 1.5 million new infections occurring per year. Approximately 20–30% of all HCV patients will develop advanced liver fibrosis, or Cirrhosis^1^. Cirrhosis is the 4th leading cause of chronic disease and results in about 1 million deaths annually^2^. Apart from hepatic decompensation resulting from cirrhosis, the other feared outcome is hepatocellular carcinoma (HCC). About 2–7% of cirrhotic patients develop HCC annually compared to 7/100,000 in the healthy population.

**Figure 1:**
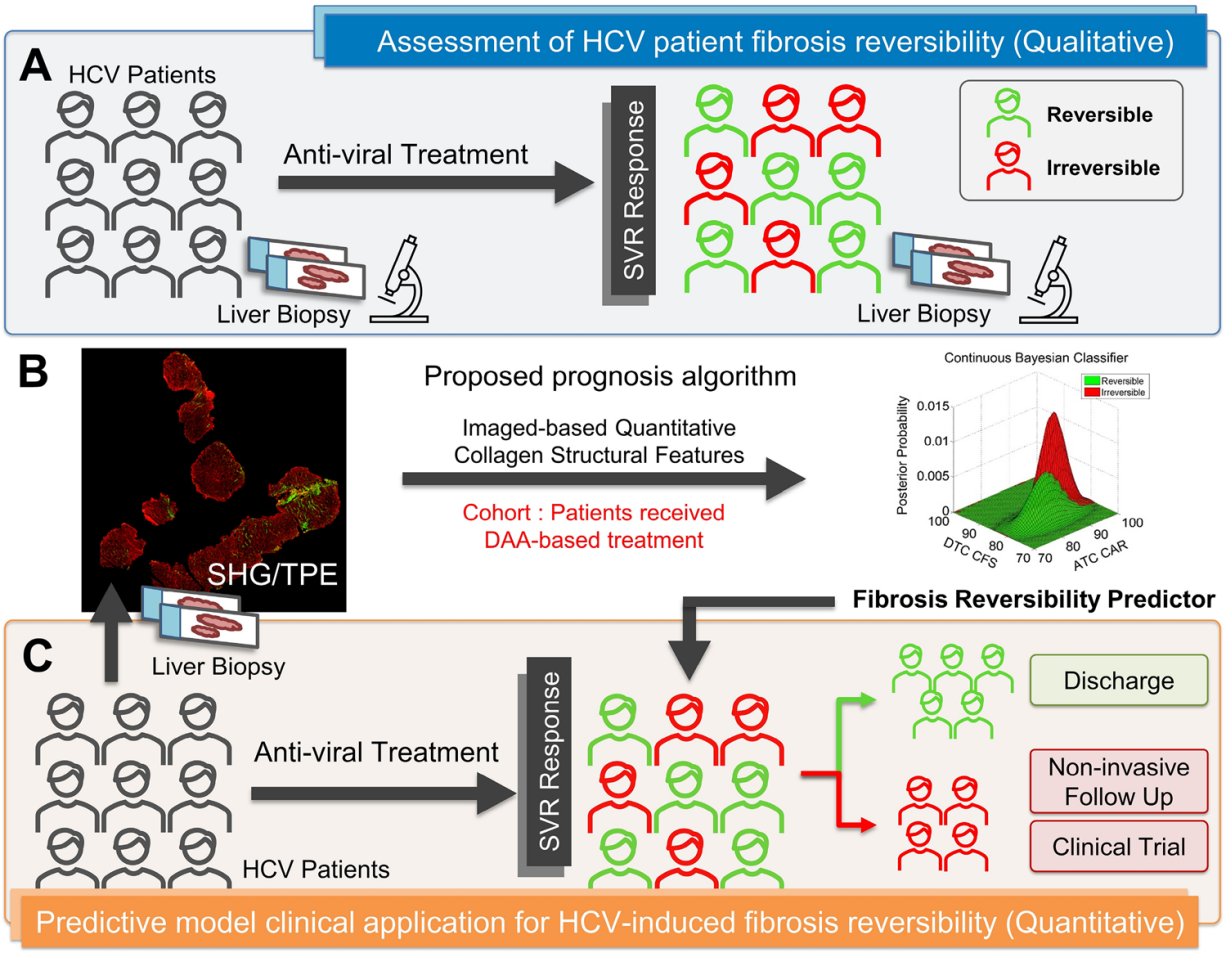
Prediction of HCV-induced liver fibrosis reversibility after SVR has significant clinical value. (**A**) HCV patient liver biopsies were taken pre-SVR and post-SVR for pathologists to assess the fibrosis reversibility. Currently, we lack efficient prognostic features that can stratify the patients for the appropriate interventions. (**B**) Liver biopsy images were scanned using TPE and SHG techniques and then processed using in-house computational algorithms. The features were selected to prototype a predictive model to classify the patients. (**C**) The desired predictive model stratifies the patients as either irreversible or reversible, indicating which patients can be discharged, retained for non-invasive follow up, or enrolled in a clinical trial.

The traditional treatment options for HCV include Peginterferon, Ribavirin and Harvoni which are associated with reversion of liver fibrosis^3^. In the past years, a few novel HCV therapeutics approved by FDA include Epclusa^®^ (Sofosbuvir) and OLYSIO^®^ (Simeprevir)^4–6^. Although Direct-Acting Antiviral agents (DAAs) report a high ratio of sustained virologic response (SVR), where HCV is no longer detected in blood plasma 24 weeks from the completion of anti-viral therapy^6^, about 30% of the cases have been reported where fibrosis has not improved post-SVR^7^ which we classify as the irreversible group shown in Fig. 1A. The process of hepatic fibrogenesis is complex and involve many actors. Hepatic stellate cells (HSCs) are known to have a pivotal role^8–10^. Tumor necrosis factor α (TNF-α)^11^, transforming growth factor β (TGF-β)^12–15^, IL-11^16,17^, and OSM^18,19^ are some of the known cytokines involved in the fibrogenesis pathways. Immune cells, such as NK cells have antifibrotic properties, and are also associated with fibrosis reversibility^20^. A recent work discovered that besides inflammation and the immune response, angiogenesis may also have a key role^21^. Stromal collagen remodeling occurs with collagen realignment in the liver parenchyma^22–24^. Several studies showed that the reduction of fibrous scar and inactivation apoptosis of myofibroblasts are also associated with fibrosis regression^25–28^. However, while the aforementioned mechanisms have been explored, the role of collagen structural remodeling in fibrosis reversibility remains lacking in literature. Biopsy sections are still the gold standard to reveal the tissue level structural morphology and provide an immense trove of information for the future research. There is some debate as to the necessity of biopsy sampling given the accuracy of non-invasive techniques, while the vast amount of information available from biopsies are undeniably valuable to further enhance the future non-invasive solutions.

In our study, liver biopsy was taken both pre- and post-treatment for clinician to assess whether the degree of fibrosis is decreased or not after treatment, as shown in Fig. 1A. In order to predict the likelihood of fibrosis reversibility of patients post-SVR, there is a need for a combined quantitative–qualitative solution to select the collagen features to help stratify them in the clinic for optimized medical interventions as shown in Fig. 1B,C. The lack of predictive features for HCV-induced liver fibrosis reversibility is mainly due to the previous imaging technical limitations. The remodeling and quantification of collagen have historically been studied using biochemical staining, such as Picrosirius Red (PSR) or Masson trichrome (MT). However, this analysis is highly dependent on the staining protocol and color deconvolution algorithms thus limiting their consistency. Such methods have an inadequate ability to identify predictive features of HCV-induced liver fibrosis reversibility, and instead only a count of the total amount of collagen in tissue samples is usually assessed, known as the Collagen Proportion Ratio (CPR). As such, the quantitative structural information of collagen fibers remains insufficient for deeper analysis that can provide predictive value on fibrosis reversibility outcomes.

While structural information of collagen is not easily accessible using traditional imaging techniques or protocols, it is central to gaining more insights into the mechanism of liver fibrosis reversibility. Recent advances in the medical imaging techniques, Two-Photon Excitation (TPE) and Second Harmonic Generation (SHG) imaging system, allow us to acquire and quantify the collagen structures in greater detail. SHG is a multiphoton, laser-based, quantitative nonlinear optical imaging technique used to identify fibrillary collagen in stain-free formalin-fixed, paraffin-embedded (FFPE) tissue. Due to its physical principles, it is highly sensitive to changes in collagen fibril, fiber structure and the remodeling of connective tissue. The application of this new image technique in the diagnosis^29–36^ and prognosis^37,38^ of hepatitis B virus (HBV) and HCV have been reported using both animal models and human clinical samples.

Drug developments of new antifibrotic medications will rely on accurate, reliable and quantitative evaluation of liver fibrosis^8,24^. Approximately eight different anti-hepatic fibrosis compounds are under development and validation at various clinical trial stages; however, there are yet to be available effective FDA-approved medicines to prevent liver fibrosis progression. After the effective antiviral treatment of HCV, the next essential need is to predict reversible vs. irreversible liver fibrosis patients for closer active surveillance and enrolment into suitable ongoing clinical trials, as shown in Fig. 1C.

**Figure 2:**
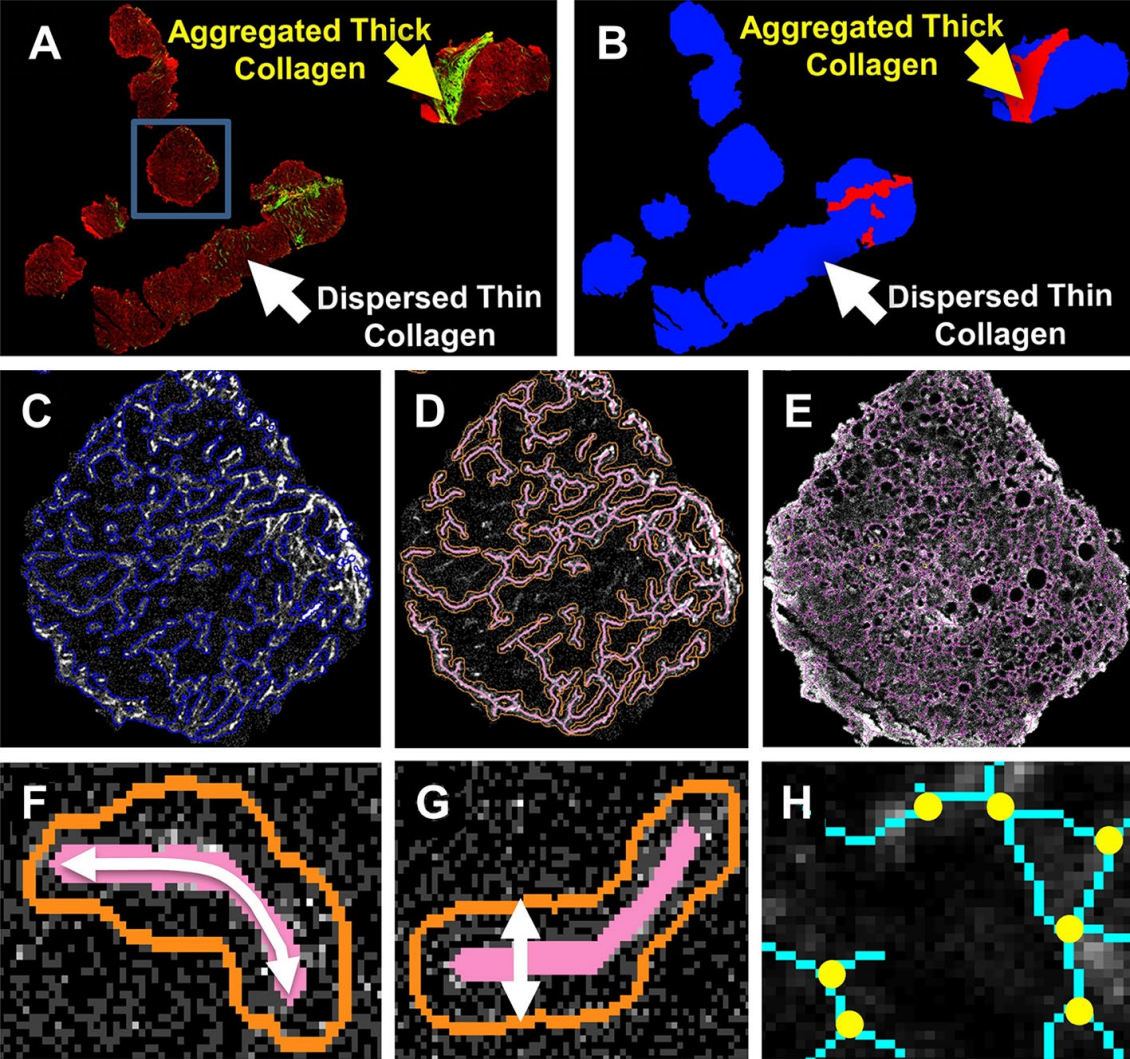
Quantitative collagen structural features extracted from automatic image analysis. (**A**) Representative TPE (red) and SHG (green) images, which specify the collagen structures. The collagen has two distinct models: aggregated thick collagen (ATC) and dispersed thin collagen (DTC). The ATC area contains highly concentrated and aggregated collagen fibers as indicated by the yellow arrow. The collagen in the DTC area is evenly and sparsely distributed as shown by the white arrow. The blue box indicates the area depicted in parts (**C**–**E**). (**B**) Our intensity-morphology based filter is able to differentiate these two types of regions successfully as shown by the yellow and white arrow. (**C**) The collagen signal is detected using a GMM model, and their connectivity is preserved for a better structural description. (**D**) Skeletonization was applied to the collagen binary image in order to identify the central line of the fiber network. (**E**) Similar solutions are applied to the TPE channel, and we extract the features from TPE accordingly. (**F**) Illustration of collagen fiber length (CFL). (**G**) Illustration of collagen fiber thickness (CFT). (**H**) Junction points detection (yellow dots) to measure the collagen network complexity.

In this study, we identified image-based quantitative prognostic features and use them to predict the risk of fibrosis reversibility in patients caused by HCV post-SVR. We provided a novel computational solution to categorize collagen into two distinct modes based on fiber SHG signal intensity and morphology: Aggregated Thick Collagen (ATC) and Dispersed Thin Collagen (DTC) (Fig. 2A,B). The differentiation of ATC and DTC provides a unique insight into collagen remodeling during liver fibrosis progression. With the use of TPE and SHG imaging techniques, we also employed a fully automatic digital collagen profiling platform developed in a previous study^39^ to quantify collagen structures using four categories of features: intensity/area, textural, structural and fiber distribution features. Finally, we used our data to infer the general processes that might underlie fibrosis reversibility.

## Results

### Patient cohort and dataset

A total of 114 images of paired pre-SVR and post-SVR biopsies were collected from 57 patients. Of the original 64 participants, six were disqualified due to incomplete information. Another patient was excluded due to virus genotype mutation and absence of SVR. Clinical blood analyses confirmed that all other patients had achieved an SVR and had completely eradicated the virus. The 29 male and 28 female patients were between 18 and 61 years of age (mean age, 44.27 ± 10.99 years). Histological fibrosis stages of each patient for both pre-SVR and post-SVR biopsies were independently and blindly reviewed by a panel of pathologists using Masson trichrome stained slides according to the Ishak scoring system^40^ in parallel. In the fibrosis assessment of biopsy specimens, paired Ishak scores are presented (pre-SVR and post-SVR) as the reference standard. Subsequently, each patient’s fibrosis status was classified into the following two groups: (i) “Reversible”: Fibrosis is reversed, Ishak score is decreased; and (ii) “Irreversible”: Fibrosis degree and Ishak score remain the same or increased.

According to independent and blinded pathological assessment of fibrosis stage, 21 patients (13 males and 8 females) improved post-SVR (“Reversible” group), and 36 patients (16 males and 20 females) developed a same or worsening of fibrosis (“Irreversible” group).

### Collagen classification into two modes: Aggregated Thick Collagen and Dispersed Thin Collagen

It is a traditional oversimplification to characterize collagen remodeling as a simple increase or decrease in total collagen content. Novel insight into the differences in collagen structures between various regions is crucial to understand the mechanism of liver fibrosis reversibility. Due to the technical limitation, the degree/extent of collagen aggregation is historically absent in the contect of fibrosis studies. We provided a novel computational solution based on fiber SHG signal intensity, texture and morphology to classify the collagen compartments into two distinct modes: Aggregated Thick Collagen (ATC) and Dispersed Thin Collagen (DTC). The original TPE (red channel) and SHG (green channel) signals of a representative image are shown in Fig. 2A. ATC area is the region containing highly concentrated and aggregated collagen fibers indicated by the yellow arrow in Fig. 2A. Our classification solution is able to distinguish the ATC area successfully, as shown by the yellow arrow in Fig. 2B. On the other side of the image, the collagen is evenly and sparsely distributed in the DTC area, which is highlighted by the white arrow in Fig. 2A,B.

### Image analysis and feature extraction

The extracted features of a smaller piece of tissue within the blue square in Fig. 2A is amplified and shown in Fig. 2C–E. Based on the two mode Gaussian Mixture Model (GMM), we detect the area occupied by the collagens and preserve their connectivity shown in Fig. 2C. Collagen Area Ratio (CAR) was measured based on the ratio of the red and blue regions within the area of the collagen binary image (ATC area and DTC areas, respectively, Fig. 2B) to the total collagen area (ATC CAR and DTC CAR, respectively). Compared with DTC areas, the collagen often occupies proportionately more area in ATC areas, where collagen is more aggregated and denser. According to our data, ATC CAR is often > 80% while DTC CAR is generally < 20%.

In Chronic HCV, the fibrosis process is portal based. It starts with portal fibrosis, periportal fibrosis, bridging septa between portal-portal, portal-central and central-central regions, and ultimately, cirrhosis. Portal tracts with their original native collagen are incorporated into most or at least many ATC areas. Skeletonization was applied to the collagen binary image in order to identify the central line of the fiber network, as shown by the red solid line in Fig. 2D. A similar approach is also applied to the TPE channel, and the results are shown in Fig. 2E. The structural features, such as Collagen Fiber Length (CFL) illustrated in Fig. 2F and Collagen Fiber Thickness (CFT) in Fig. 2G, were calculated accordingly. The junction points of the collagen network were then identified as the yellow dots in Fig. 2H. The density of the junction points quantifies the complexity of the collagen or tissue networks. Supp. Table 1 provides a non-comprehensive list of the key features we extracted together with their definitions. Similar to ATC CAR and DTC CAR, all other extracted features are calculated within the ATC area and DTC area separately. For example, CFL will have ATC CFL and DTC CFL.

### Evaluation of quantitative collagen structural features

We performed univariate analyses for feature selection based on t-test evaluation to assess the prognostic value of each individual collagen feature for HCV fibrosis reversibility. The ATC Area Ratio is a measurement to quantify the relative amount of given tissue is occupied by the aggregated collagen, defined as the ratio between the area of red region in Fig. 2B and the total tissue area (the combination of red and blue regions). ATC Area Ratio is distinct from ATC CAR since ATC area is generally not fully occupied by collagen compartments, whereas ATC CAR is measured only within collagen areas.

**Figure 3:**
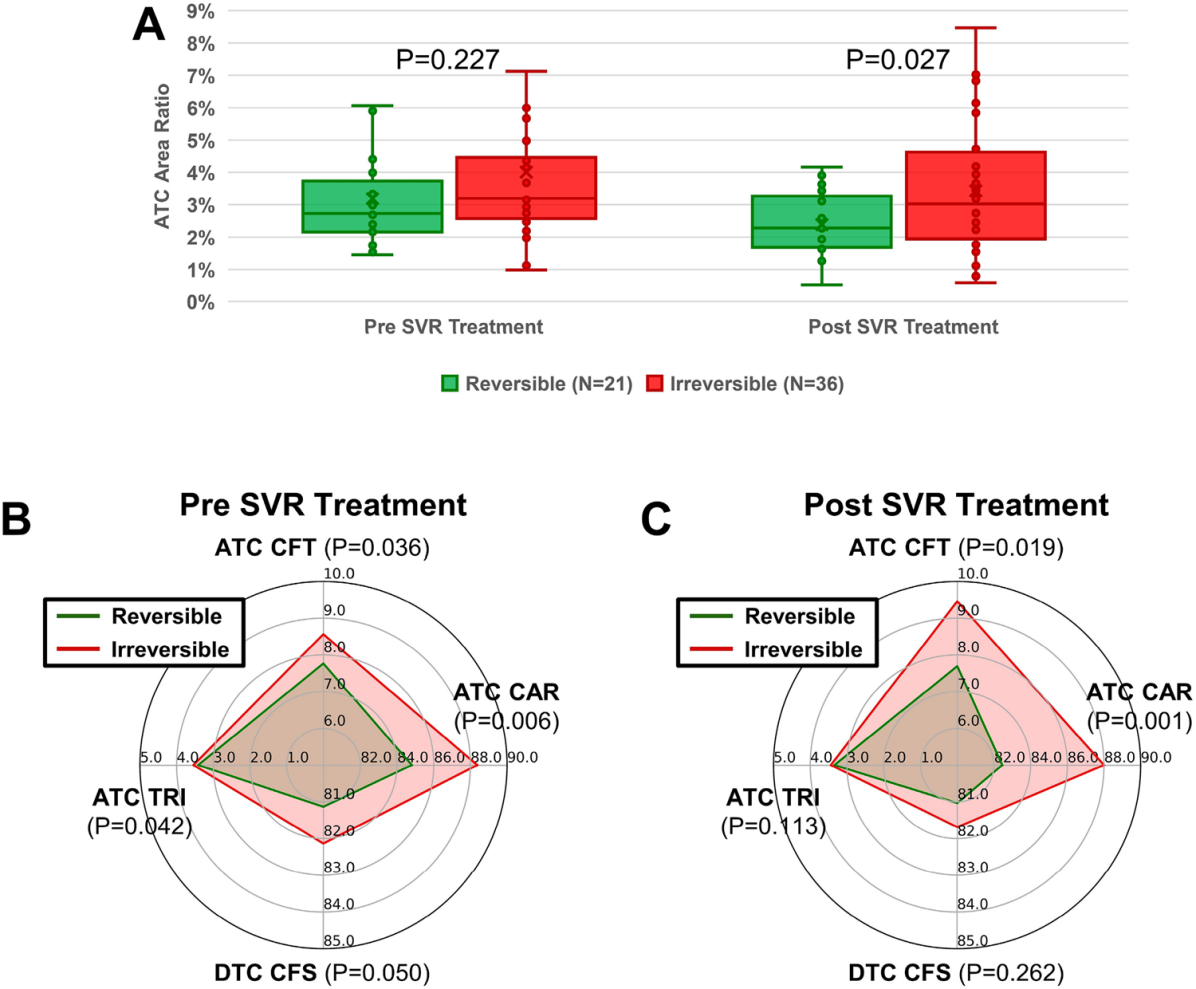
Evaluation of the extracted quantitative collagen structural features between the reversible and irreversible patient groups. (**A**) The statistical significance is not shown in ATC Area Ratio of reversible and irreversible groups pre-SVR treatment. After SVR Treatment, ATC Area Ratio of both groups decreased and significant difference is observed. (**B**) Four selected features with statistical significance, named ATC CAR, ATC CFT, ATC TRI and DTC CFS, between reversible (green line) and irreversible (red line) patient groups pre-SVR treatment. (**C**) The profiles defined by these four given features of the reversible (green line) and irreversible groups (red line) post-SVR treatment.

Our data shows that ATC Area Ratio has almost no correlation with Ishak scores for the pre-SVR and post-SVR biopsies in the reversible group given R = 0.152 and R = 0.133, respectively in Supp. Fig. 1A,C. However, for the irreversible group, there is a stronger correlation between ATC Area Ratio and Ishak scores of the pre-SVR and post-SVR biopsies given R = 0.688 and R = 0.477, respectively Supp. Fig. 1B,D. This indicates that collagen aggregation plays a key role in fibrosis reversibility. Although the reversible and irreversible cases do not demonstrate the statistical significance of ATC Area Ratio pre-SVR, the ATC Area Ratio has a statistically significant difference between the two groups of patients post-SVR (Fig. 3A). This result indicates that SVR will reduce the ATC Area Ratio of both reversible and irreversible cases, but more significantly in the reversible group, supporting the view that collagen aggregation is one of the key factors that affect fibrosis reversibility.

Next, we analyzed the quantitative structural features extracted from ATC and DTC regions. Four features (ATC CAR, ATC CFT, ATC TRI and DTC CFS) demonstrated significant differences associated with the reversibility of HCV fibrosis (Fig. 3B). As shown in the figure, Collagen Area Ratio (CAR) is one of the fundamental features measuring the abundance of collagen in the liver tissue. From a practical understanding, portal-based, aggregated fibrosis with septa formation is the characteristic pattern of fibrosis in chronic HCV livers; hence, a change in ATC CAR was to be expected. However, perisinusoidal and dispersed fibrosis is usually not an issue in HCV cases, unlike in NAFLD/NASH cases; hence, no significant change in DTC was anticipated. Indeed, ATC CAR showed a significant difference between reversible and irreversible cases (p = 0.006) (Fig. 3B), while DTC CAR did not (data not shown). The ATC CAR values are correlated with Ishak scores for both reversible (R = 0.509) and irreversible cases (R = 0.654) pre-SVR (Supp. Fig. 2A,B), while the correlation coefficient decreases for both reversible and irreversible groups post-SVR (Supp. Fig. 2C,D). This indicates that ATC CAR has predictive value in the prognosis of liver fibrosis reversibility between the two patient groups. The risk of irreversibility is higher when a patient has more collagen in the aggregated mode, i.e. ATC CAR is higher.

In the ATC regions, Collagen Fiber Thickness (ATC CFT), a measure of the girth of each collagen fiber, is also higher in the irreversible than the reversible cases (Fig. 3B) (p = 0.036). This result is consistent with the higher ATC CAR found in irreversible cases; it is intuitive that if the collagen fiber is thicker, it tends to occupy more area. We did not observe any significant difference between the two groups in DTC CAR; however, we noticed that the collagen fibers in the DTC mode are straighter, i.e. DTC collagen fiber straightness (DTC CFS) is higher, in the irreversible HCV group compared with the reversible group (Fig. 3B) (p = 0.050). In general, thicker collagen fiber in the ATC region and straighter collagen in the DTC suggest less likelihood of fibrosis reversibility. This structural difference in these different collagen modes implies that fiber packing is different in reversible and irreversible cases.

One significant feature extracted from the TPE channel between the two groups is shown in Fig. 3B. In the ATC area, we found that Tissue Reticular Index (ATC TRI), a measurement of the tissue structural complexity, is significantly higher in the irreversible group’s samples, suggesting that tissue structures form a more complex network in this group. The increase in ATC CAR is associated with higher ATC TRI, although the mechanism is not clear at this juncture. We next explored the change of these four features post-SVR (Fig. 3C). After SVR, ATC TRI and DTC CFS both lose their significance; however, the difference between ATC CAR and ATC CFT is dramatized, i.e. p-value further decreased. For example, the p-value of ATC CAR decreased from p = 0.006 to p = 0.001. We conclude that the reversible and irreversible groups have significant difference pre-SVR (Fig. 3B), and their responses in collagen structure post-SVR are also distinct (Fig. 3C).

### Relative changes of quantitative collagen and tissue structural features pre-SVR and post-SVR

**Figure 4:**
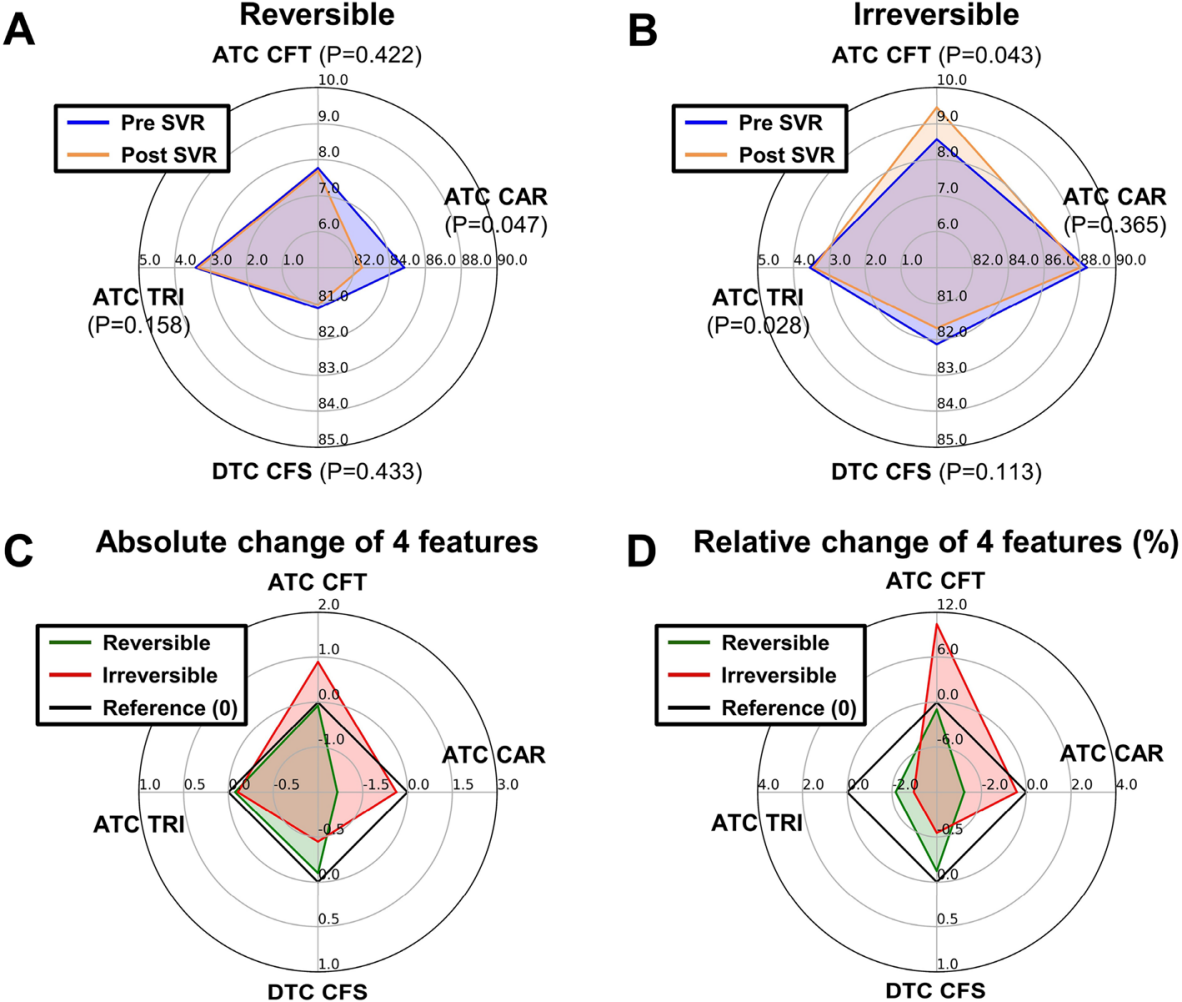
The relative changes of collagen features in reversible and irreversible patient groups pre-SVR and post-SVR. (**A**) The features of reversible patient were slightly improved, i.e. shrank from the blue line (pre-SVR) to the yellow line (post-SVR), especially for ATC CAR, which shows statistical significance. (**B**) The irreversible group’s profile is noticeably different from that of the reversible group. The ATC CFT increased and the ATC TRI decreased post-SVR with statistical significance. The decrease of ATC TRI is potentially associated with the increase of ATC CFT since the larger area occupied by the collagen might lead to a simpler network of the cellular tissue. (**C**) The absolute differences of the reversible (green line) and irreversible (red line) groups’ profile pre-SVR and post-SVR. (**D**) The relative differences in percentage of the reversible (green line) and irreversible (red line) groups’ profile pre-SVR and post-SVR.

To further understand the relative changes between the two patient groups pre-SVR and post-SVR, we present a comparison of our data in Fig. 4. Although there are some structural differences for the reversible group, the differences are not statistically significant except for ATC CAR (Fig. 4A). The small differences in the four features pre-SVR and post-SVR indicate that the fibrosis of reversible group is slightly improved and does not worsen. The irreversible group’s results pre-SVR and post-SVR are presented in Fig. 4B. The fiber girth as measured by the ATC CFT shows a clear trend of increase post-SVR with clear significance. Another significant feature for the irreversible group is the structural complexity as measured by the ATC TRI. The decrease of ATC TRI might be associated with the increase of ATC CFT since more area is occupied by the collagen, which may cause a simpler network of the cellular tissue, or vice versa. However, we did not observe the same result in the reversible group (Fig. 4A). Further investigation is required to establish the physiological mechanisms behind ATC TRI decreasing and ATC CFT increasing for the irreversible group.

The absolute differences and relative changes in percentage of those four features pre-SVR and post-SVR are presented in Fig. 4C,D. In general, for the reversible group, all four features remain the same or are slightly improved, as shown by the green line in Fig. 4C,D, compared with their baseline values (black line), while for the irreversible group, the ATC CFT has clearly increased, which may be associated with the unknown mechanism of fibrosis reversibility.

### Prognostic features selection and the optimization of predictive models

Based on our patient cohort and collagen structural analysis, the above four quantitative image-based collagen structural features show promising prognostic value of HCV-induced liver fibrosis reversibility. It is critical to understand the dependence of the features and build an optimized predictive model. In the ATC area, there are two significant features: collagen area ratio (ATC CAR) and collagen fiber thickness (ATC CFT). In general, higher ATC CAR and ATC CFT indicate higher risk of irreversibility even after SVR. However, ATC CAR and ATC CFT are not completely independent. As shown in Supp. Fig. 3A, the linear relationship between these two features shows a strong correlation as indicated by the black dotted line. The increase in ATC CFT post-SVR reveals that collagen fibers became thicker in the irreversible group, whereas ATC CFT remains almost the same for the reversible group. This implies that the collagen packing pattern is different in the two groups. In the DTC area, the collagen fiber straightness (DTC CFS) has significant predictive value for fibrosis reversibility. The structural complexity measurement (ATC TRI) extracted based on the two-photon excitation (TPE) channel is also important, although the mechanism needs further exploration, and we did not include it in our predictive model while worth to further investigate.

The three collagen features, i.e. ATC CAR, ATC CFT and DTC CFS, have the potential to build a practical predictive model for clinical use. Based on our data thus far, the aggregated collage area ratio and dispersed fiber straightness (ATC CAR and DTC CFS, respectively) form the selected two prognostic features with predictive value of liver fibrosis reversibility in HCV patients since both metrics are significantly higher in the irreversible group.

The significant statistical differences between the selected features of pre-SVR biopsies enable us to prototype a predictive model of fibrosis reversibility. Such predictive models are essential for clinical practice to stratify patients in the healthcare system and select suitable patient candidates for clinical trials. To optimize our predictive model building, we used three different methods to build a clinical predictive model for HCV-induced liver fibrosis reversibility: (1) Bayesian model, (2) Support Vector Machine (SVM) and (3) Relevance Vector Machine (RVM).

To optimize our predictive model, we evaluated different methods according to their performance and visualized decision boundary in the 2D feature space. The models based on SVM and RVM methods are presented in Supp. Figs. 4, 5. Although SVM model in Supp. Fig. 5A,C achieved the best performance, the complicated decision boundary in 2D feature space may indicate a rick of overfitting due to limited sample number. RVM model is also not ideal due to its poor specificity.

**Figure 5:**
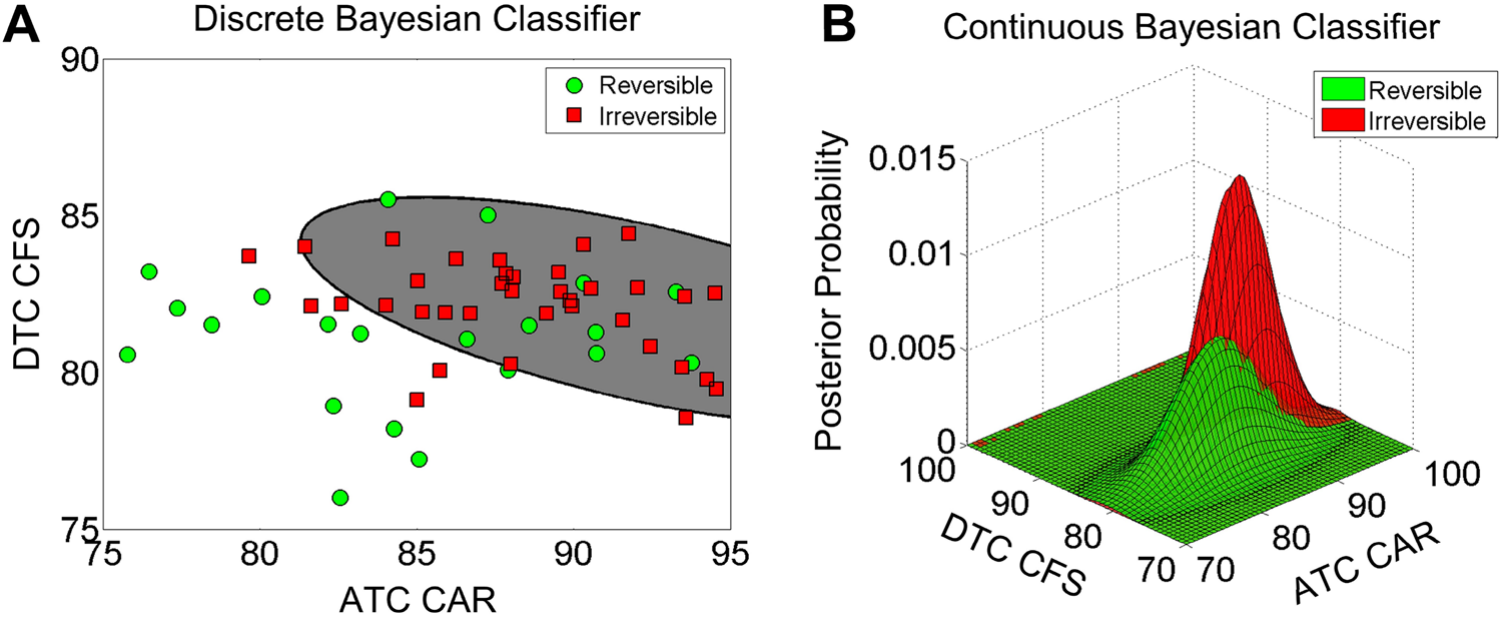
The discrete and continuous Bayesian predictive models for the HCV induced liver fibrosis reversibility. (**A**) The optimized discrete model using two selected features, ATC CAR and DTC CFS, and their distribution in the 2D feature space. (**B**) The optimized continuous model using ATC CAR and DTC CFS and their probability distribution function in the 2D feature space.

The optimal Bayesian model is presented in Fig. 5, which indicates that the 57 patients can be divided into two groups based on ATC CAR and DTC CFS. In Fig. 5A, the green dots represent the reversible cases, and the red squares represent the irreversible cases. The number of patients with reversible and irreversible fibrosis in each group is also moderately well-balanced, i.e. 21 reversible cases vs. 36 irreversible cases. In general, a higher ATC CAR value and greater DTC CFS are indicative of poor prognosis, whereas a lower ATC CAR value and lesser DTC CFS are indicative of good prognosis (i.e. reversible fibrosis). The two-feature decision boundary is represented by the black solid line between the irreversible (gray) and reversible (white) domains in Fig. 5A. Figure 5B represents the continuous Bayesian predictive model based on ATC CAR and DTC CFS. The red surface indicates the irreversible while the green surface indicates the reversible groups respectively.

The performance metrics of the discrete and continuous model is presented in Table 1. Based on both the discrete and continuous models, we can successfully identify ~ 85% of irreversible cases and about 62% of reversible cases. The selected quantitative features and Bayesian predictive model have a potentially high clinical prognostic value for HCV-induced liver fibrosis reversibility prediction.

**Table 1:** The performance metrics of the discrete and continuous Bayesian predictive model.

Measurement	Discrete model	Continuous model
Recall (sensitivity)	83.33%	84.22%
Specificity	57.14%	61.15%
Precision	76.92%	68.51%
F1 score	80.00%	75.56%
Accuracy	72.41%	72.71%

## Discussion

Certainly, a larger sample size of patient data is desired to further validate a predictive model suitable for immediate clinical application, while such paired liver biopsy samples are valuable and rare. The features we identified show promising results with limited data and we used the selected features to develop a proof-of-concept model, prototyping three different predictive models to examine the prognostic value of our identified features. Based on our analysis, we were able to achieve ~ 85% sensitivity in stratifying irreversible cases with pre-SVR liver biopsy by employing the prototyped predictive model, as shown in Table 1. However, the two-feature model is less specific for negative patients, i.e. about 62% of reversible cases are successfully detected, consistent with the uncertainty of pathology assessment. The strength of certainty in the predictive model can reliably inform clinicians on the level of patient surveillance required and to allow for appropriate early intervention, if necessary, since predictions can be made based on a single pre-SVR biopsy. For the truly negative patients (reversible group), we can greatly space out follow-up visits. Reversible cases identified with less confidence can be stratified together with the irreversible cases for active surveillance using non-invasive techniques as follow-up.

Several avenues for further research have been identified in our study. The significant difference of ATC CFT between pre- and post-SVR implies that the collagen packing pattern is also potentially different in the two groups. It may need another imaging modality, such as Electrical Microscope (EM), with higher resolution to further confirm our discovery, while there are some technical challenges to identify the regions of ATC and DTC regions in EM images. In the dispersed, thin collage (DTC) area, the collagen fiber straightness (DTC CFS) has significant predictive value for fibrosis reversibility too. In future, it is also worth exploring the DNA and RNA variation between these two groups of patients to understand their role in the collagen aggregation patterns and the packing structure of collagen. The structural complexity measurement (ATC TRI) extracted based on the TPE channel is also important, but the cause of ATC TRI difference between the two groups is not clear. Thus, we did not include unclear variable in our predictive model. It might be associated with the increase of ATC CFT and the collagen aggregation mechanism. The causality and mechanism between the collagen aggregation and tissue structural changes needs further exploration too.

Using the pre-SVR biopsies and the baseline of fibrosis scoring provided by post-SVR biopsies, we proved that it is in principle possible to develop a clinical predictive model through a larger scale study. For the reversible group, all four features are slightly decreased in most cases post-SVR. In the irreversible group, ATC CFT is notably the only feature which increased post-SVR, which might also be relevant to the decrease of ATC TRI as discussed previously. Combined with the straightness difference in DTC area, this result strongly indicates that the structure of collagen fiber packing is potentially different between the irreversible and reversible cases; further research on the arrangement and spatiotemporal interplay of these different structures between pathways and cell types involved may be key to understanding the regenerative response behind fibrosis reversibility and potentially result in novel targets for pharmaceutical treatments. The collagen fiber packing structure is potentially also associated with the degree of collagen aggregation. While our primary objective was to identify image-based prognostic features associated with fibrosis reversibility prediction, our post-SVR biopsy analyses have also revealed new avenues for mechanistic research into how fibrosis is reversed. Of note, another clinical feature, patient age, was identified is the patient age as shown in Supp. Fig. 6. Younger patients overall have less risk of irreversible fibrosis. Fibrosis in patients < 30 years of age have a higher chance of being reversible, which might be due to cellular senescence or metabolic differences between younger and elder populations.

Despite its drawbacks, the gold standard for evaluating liver fibrosis is still the liver biopsy. Some of the main drawbacks of a biopsy in clinical diagnosis include its cost and potential risk of patients. In this regard, the confluence of invasive and non-invasive methods along with the developing field of medical imaging computer assisted intervention may help alleviate the problems faced by biopsy techniques such as sampling error. Non-invasive tools include serologic methods. There are various serologic approaches with different advantages and disadvantages; these methods are mainly employed in limited medical resource communities and regions. The elasticity-based imaging technique, such as Fibroscan, has become an effective non-invasive assessment tool for liver fibrosis; however, Fibroscan results are significantly affected by the intake of food, liver inflammation, intrahepatic cholestasis, hepatic congestion and fatty liver condition^41^. As such, the reliability and accuracy of this method is reduced for patients with marked obesity and ascites. Although the above mentioned serologic methods have good performance in excluding and/or identifying patients with mild or severe liver fibrosis, the accuracy of intermediate liver fibrosis remains unsatisfactory which limits its effectiveness as a stand-alone test. Hence, while an initial, pre-SVR liver biopsy may be unavoidable for some instances, a reliable predictive tool that can indicate the reversibility of fibrosis based off a single pre-SVR biopsy can greatly reduce the need for a second, post-SVR biopsy—especially in conjunction with a suitable non-invasive sampling technique which can be used to complement the predictive model with active surveillance.

While the technology for non-invasive techniques have become increasingly effective, none of these approaches can obtain the completeness of collagen structural information at tissue level required to indicate whether the fibrosis observed is irreversible or reversible. Such information is untapped information that future researchers may make use of to further improve non-invasive screening. Our work revealed the amount of ATC components are different between reversible and irreversible patients. The stiffness of ATC components in the liver is potentially higher than the DTC regions. Applied to the clinical setting, an accurate biopsy has the potential to provide a tell-tale certainty to a patient’s condition if advances were made in enhancing specificity. The reality is that both technologies have can benefit from dual development through the advancement of the other. In the case that future non-invasive scanning solutions have better spatial resolution and consistence to detect the different stiffness of different part of liver, i.e. measure the ATC volume in a non-invasive way as we discovered, it will be possible to explore its clinical application to predict the risk of reversibility using the non-invasive screening approaches.

Although the findings in this work are promising for the stratification of irreversible patients in healthcare system, we must emphasize that it is still preliminary and not yet replicated using an independent larger cohort. Well-designed rigid prospective clinical validations/trials must be conducted to provide much needed confirmation of the model’s performance before its clinical application. As time either improves or worsens the features associated with liver fibrosis, early identification and stratification of HCV patients into reversible and irreversible liver fibrosis has significant clinical value as both groups can be effectively managed, monitored and treated appropriately to improve clinical outcomes.

## Methods

### Ethical approval

The Institutional Review Board (IRB) of Peking University People’s Hospital provided ethical approval for the use of patient material (IRB Ref: 2017PHB133-01). Chronic HCV patients received DAA-based treatment for 12 weeks or 24 weeks. Written informed consent was obtained from each patient, and the study was carried out in accordance with the approved guidelines.

### Clinical inclusion/exclusion criteria

The study cohort consisted of paired liver specimens from patients with chronic hepatitis C virus infection, who were admitted to the Peking University Hepatology Institute, Peking University People’s Hospital between March 2014 and December 2016. Clinical and pathological characteristics were obtained from the patients’ medical records.

Key inclusion criteria were as follows: aged 20–70 years; chronic hepatitis C infection with or without cirrhosis; HCV-RNA levels > 10,000 IU/mL at baseline; received antiviral treatment with direct-acting antiviral agents (DAAs) for 12 or 24 weeks; achieved a sustained virologic response (SVR; named cure of hepatitis C virus infection) by 24 weeks after treatment completion. Paired liver biopsies performed at both baseline and week 24 after treatment.

Exclusion criteria were as follows: co-infection with hepatitis B virus or human immunodeficiency virus (HIV); the presence of other forms of chronic liver disease (including alcoholic liver disease); decompensated liver diseases (including ascites, variceal bleeding, or hepatic encephalopathy); alpha-fetoprotein > 100 ng/mL or creatinine clearance rate < 50 ml/min; any malignant tumor; any complications causing severe heart, lung, kidney or brain problems; diabetes; blood or other notable systematic diseases; BMI > 28 kg/m^2^; severe neurological or psychological disease; pregnancy; lactating women.

### Biopsy tissue sample preparation

Archived formalin-fixed, paraffin-embedded (FFPE) liver biopsy tissue samples were obtained from 64 patients diagnosed with HCV infection at the Hepatology Institute, Peking University People’s Hospital from 2014 to 2016. Clinicopathological features of patients were collected and summarized in Table 2. The core biopsies were at least 10 mm in length and the diameter is 0.84 mm. Unstained, 4–5 μm thick histological sections were prepared for SHG-imaging and, thereafter, stained with H&E and Masson trichrome for histological assessment. Ishak staging system for fibrosis was employed (score 0—no fibrosis; 1—fibrous expansion of some portal areas, with or without short fibrous septa; 2—fibrous expansion of most portal areas, with or without short fibrous septa; 3 fibrous expansion of most portal areas with occasional portal to portal bridging; 4—fibrous expansion of portal areas with marked bridging (portal to portal) as well as portal to central; 5—marked bridging with occasional nodules (incomplete cirrhosis); and 6—cirrhosis, probable or definite)^40^. All clinical and pathological characteristics were obtained from the patients’ medical records which were irreversibly anonymized before being accessed.

**Table 2:** Demographic, laboratory and pathology data for the patient cohort.

Variable	Reversible (N = 21)	Irreversible (N = 36)	*p* value
	N (%) or median (range)		
Age (years)	29 (21–60)	48 (23–67)	0.0045
Female	8 (38.10%)	20 (55.56%)	
Male	13 (61.90%)	16 (44.44%)	
ALT	54 (20–152)	52 (4–228)	0.7442
AST	35 (24–95)	43 (16–143)	0.4125
ALB	45 (37–51)	45 (39–53)	0.6891
TBIL	10 (6–29)	11.5 (6.7–38)	0.5577
INR	1 (0.9–1.22)	1 (0.8–1.56)	0.8354
PLT	213 (109–298)	164.5 (42–299)	0.0254
Fibrosis stage (Ishak)			
0	0 (0%)	2 (5.56%)	
1	3 (14.29%)	2 (5.56%)	
2	2 (9.52%)	2 (5.56%)	
3	3 (14.29%)	8 (22.22%)	
4	8 (38.10%)	8 (22.22%)	
5	1 (4.76%)	3 (8.33%)	
6	4 (19.05%)	11 (30.56%)	

ALT: alanine transferase; ALB: albumin; AST: aspartate transferase; INR: international normalized ratio; PLT: platelet; TBIL: total bilirubin.

### Image acquisition

Parallel to clinical treatments and pathological assessment, the pre-SVR and post-SVR biopsies (after 12 months) from 57 subjects were first deparaffinized and then scanned using Histoindex Genesis 200. Each image of a given patient consists of at least one completed biopsy as shown in Fig. 2A. The Genesis Scanner acquired the image of collagen fibers (with SHG) using the green channel and the tissue structure (with TPE) using the red channel, both of 8-bit intensity with 20× objective magnification. The laser power of the system was set at “High,” and binning was set at two frames to reduce the random noise. The brightness of each pixel represents the collagen density in the fibers. The imaging protocol remains consistent through our study to ensure a quantitative image acquisition.

### Image analysis

These acquired images were processed using our designed computational image analysis pipeline between the period of January 2018 to December 2020. Each anonymous image of a given biopsy was named according to its anonymized pathological identification associated with clinical pathological features. First, the Gaussian smoothing filter is applied to preprocess the SHG channel. A sample mask was then defined to cover the area occupied by the liver tissue. Next, the collagen signal is extracted from five random image ROIs from each given image. The signal value in pixel is used to construct the collagen signal classification model using an expectation–maximization (EM) algorithm as follows: (i) A random initial guess for the model’s features is made from the “Expected” distribution (training dataset), typically known as E-step; (ii) The probability distribution from the E-step is adjusted to include the new dataset, typically known as the M-step; (iii) Estimated features can be obtained whenever the distribution does not change from E-step to M-step. The two mode Gaussian mixture model (GMM) was applied to determine whether a given pixel signal is positive for collagen. Based on the GMM model, an optimized thresholding value was determined to binarize the image, creating a collagen binary image. A similar scheme was applied to the red channel (TPE) to create a tissue binary image.

### Feature extraction and analysis

The acquired biopsy sample images comprised two distinctive regions: (i) Regions that contain highly concentrated collagen compartment (Fig. 2A, yellow arrow) and (ii) Regions that contain evenly and sparsely distributed collagen fiber (Fig. 2A, white arrow). Based on SHG signal intensity and morphology, a novel computational filtering pipeline was designed to categorize the collagen into these two distinct modes: Aggregated Thick Collagen (ATC) and Dispersed Thin Collagen (DTC). Intensity/area, textural, structural and fiber distribution features were then measured. Each feature is measured at the ATC, DTC and “ALL” (the combination of both ATC and DTC) tissue regions. A non-exhaustive list of features and their definitions are provided in Supp. Table 1. The features were classified into the following categories: texture, thickness, length, straightness and orientation. Once the features were extracted from the images, their relationships with clinical pathological features were assessed. Statistically significant associations (p < 0.05) are presented.

### Statistical analysis

There are 41 features extracted from the images of the biopsies of patients and all the features are divided into two groups, i.e. reversible and irreversible, accordingly. T-test was performed to find the most distinctive features that can differentiate between the two groups. Four features which have significant difference (p < 0.05) between two groups of patients were selected for further investigation.

## Supplementary Information

Supplementary Information.

## Data Availability

The datasets used and/or analyzed during the current study available from the corresponding author (WY) on reasonable request.
