# Supplementary material for: Quantitative image-based collagen structural features predict the reversibility of hepatitis C virus-induced liver fibrosis post antiviral therapies
Source: Sci Rep. 2023 Apr 19;13:6384. doi: 10.1038/s41598-023-33567-4 (PMC10115775; doi:10.1038/s41598-023-33567-4)
Supplement: Supplementary file 1 — Supplementary Information. [file 41598_2023_33567_MOESM1_ESM.pdf]

Supplementary File

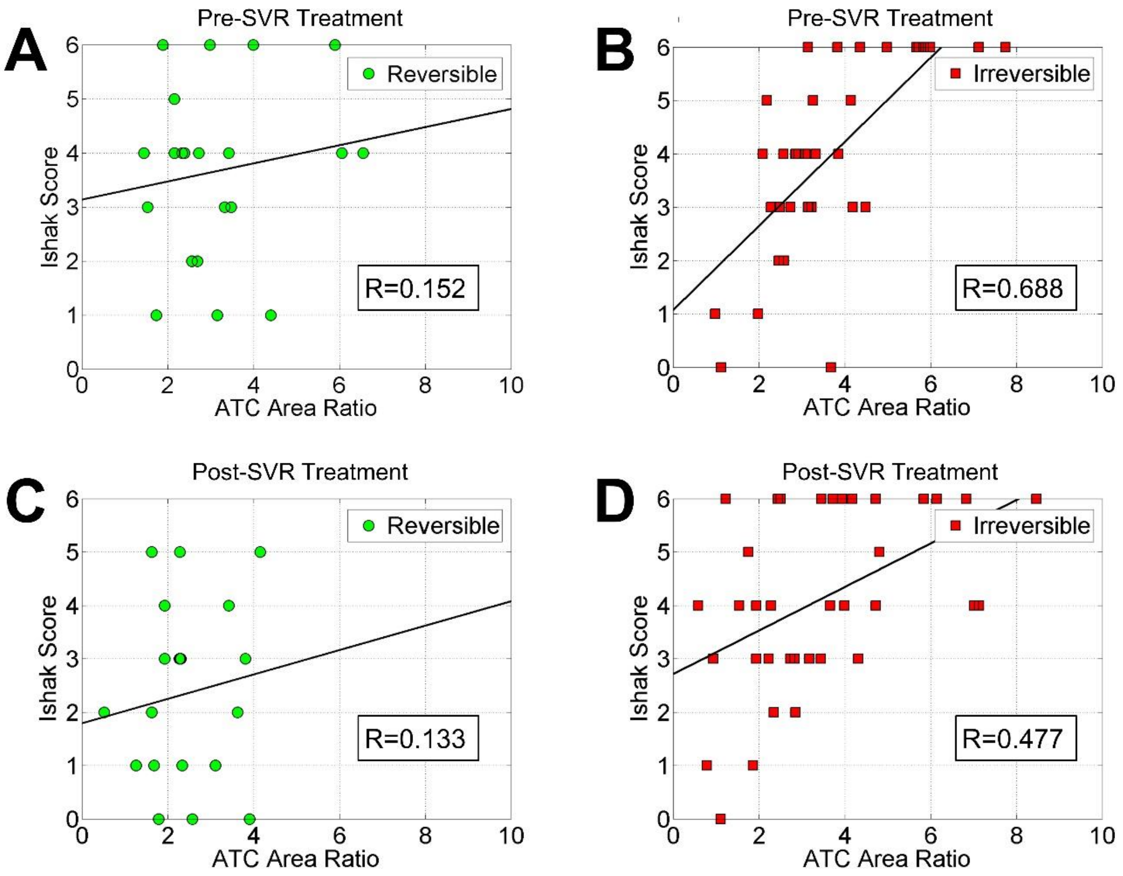

Supp. Fig. 1. Correlations of reversible and irreversible patients' ATC Area Ratio and Ishak Score pre-SVR and post-SVR treatment. **A).** ATC Area Ratio of reversible patients has almost no correlation with Ishak Score pre-SVR treatment (R=0.152). **B).** ATC Area Ratio of irreversible patients has much stronger correlation with Ishak Score pre-SVR treatment (R=0.688). **C).** ATC Area Ratio of reversible patients has almost no correlation with Ishak Score post-SVR treatment (R=0.133). **D).** ATC Area Ratio of irreversible patients has moderate correlation with Ishak Score post-SVR treatment (R=0.477), though weaker than that of pre-SVR treatment (R=0.688).

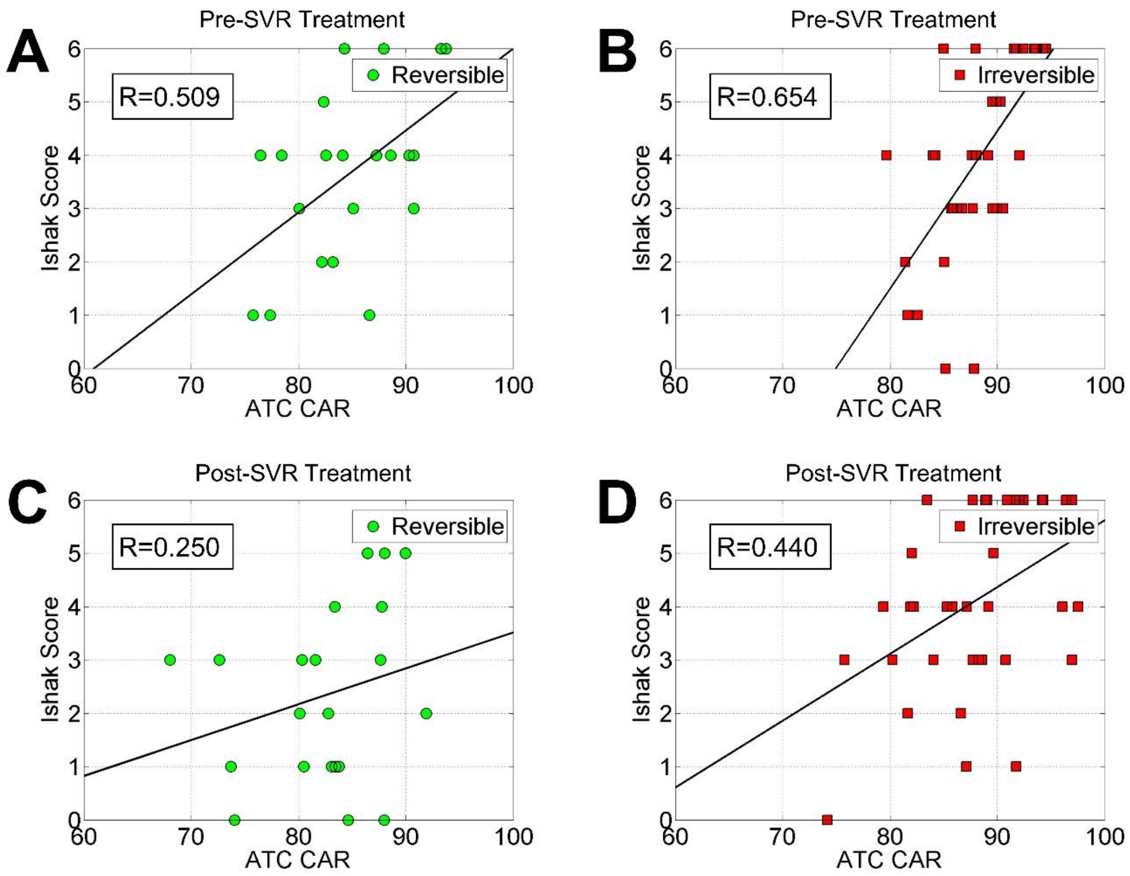

Supp. Fig. 2. Correlations of reversible and irreversible patients' ATC CAR and Ishak Score pre-SVR and post-SVR treatment. **A).** ATC CAR of reversible patients has moderate correlation with Ishak Score pre-SVR treatment (R=0.509). **B).** ATC CAR of irreversible patients has stronger correlation with Ishak Score pre-SVR treatment (R=0.654). **C).** ATC CAR of reversible patients has almost no correlation with Ishak Score post-SVR treatment (R=0.250). **D).** ATC CAR of irreversible patients has moderately strong correlation with Ishak Score post-SVR treatment (R=0.440), though weaker than that of pre-SVR treatment (R=0.654).

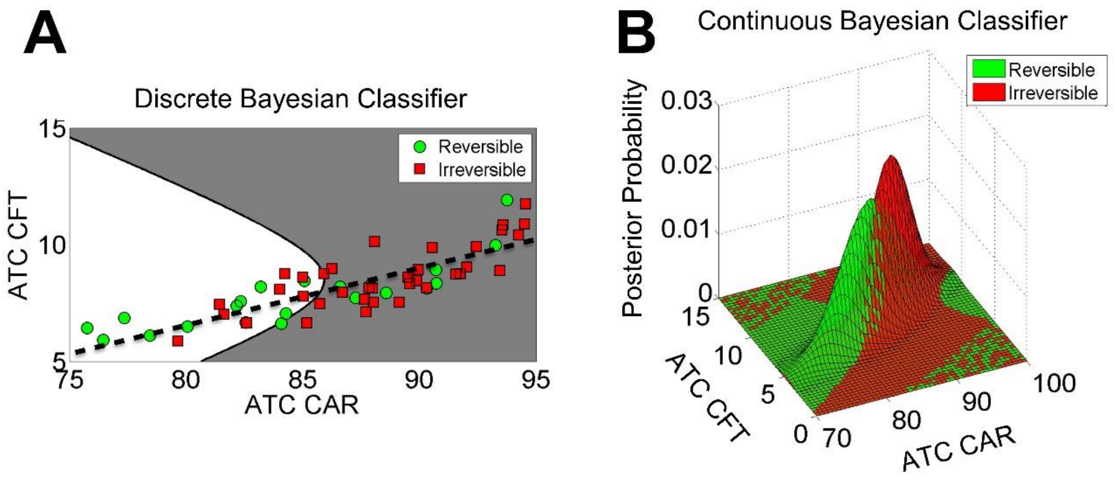

C

Recall	77.78%
Specificity	52.38%
Precision	73.68%
F1 Score	75.68%
Accuracy	67.24%

D

Recall	78.77%
Specificity	53.87%
Precision	63.15%
F1 Score	70.10%
Accuracy	66.34%

Supp. Fig. 3. Prototype of Bayesian predictive models using ATC CAR and ATC CFT. **A).** The distribution of reversible (green dots) and irreversible (red squares) patient data in the 2D feature space of ATC CAR and ATC CFT. The solid line between the white and gray domains is the decision boundary between the two groups. The data also shows that ATC CAR and ATC CFT have a strong linear correlation. **B).** The reversible and irreversible patients' probability distributions function of ATC CAR and ATC CFT. Green surface is for reversible patients, and red surface is for irreversible patients. **C).** The performance metrics of the prototyped discrete predictive model based on ATC CAR and ATC CFT. **D).** The performance metrics of the continuous predictive model based on ATC CAR and ATC CFT.

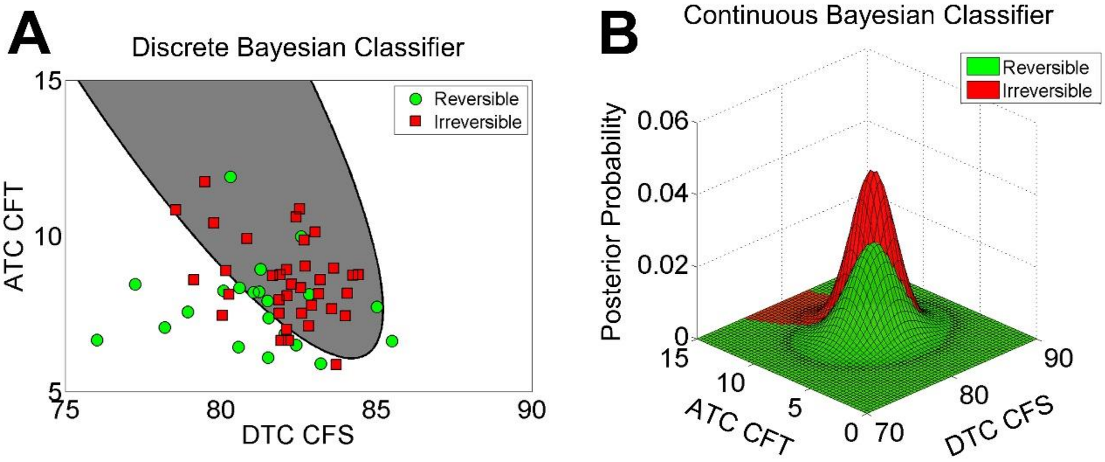

C

Recall	80.56%
Specificity	61.90%
Precision	78.38%
F1 Score	79.45%
Accuracy	72.41%

D

Recall	80.44%
Specificity	59.16%
Precision	66.32%
F1 Score	72.70%
Accuracy	69.80%

Supp. Fig. 4. Prototype of Bayesian predictive models based on DTC CFS and ATC CFT. **A).** The distribution of reversible (green dots) and irreversible (red squares) patient data in the 2D feature space of DTC CFS and ATC CFT. The solid line between the white and gray domains is the decision boundary between the two groups. **B).** The reversible and irreversible patients' probability distributions function of DTC CFS and ATC CFT. Green surface is for reversible patients, and red surface is for irreversible patients. **C).** The performance metrics of the prototyped discrete predictive model using DTC CFS and ATC CFT. **D).** The performance metrics of the continuous predictive model based on DTC CFS and ATC CFT.

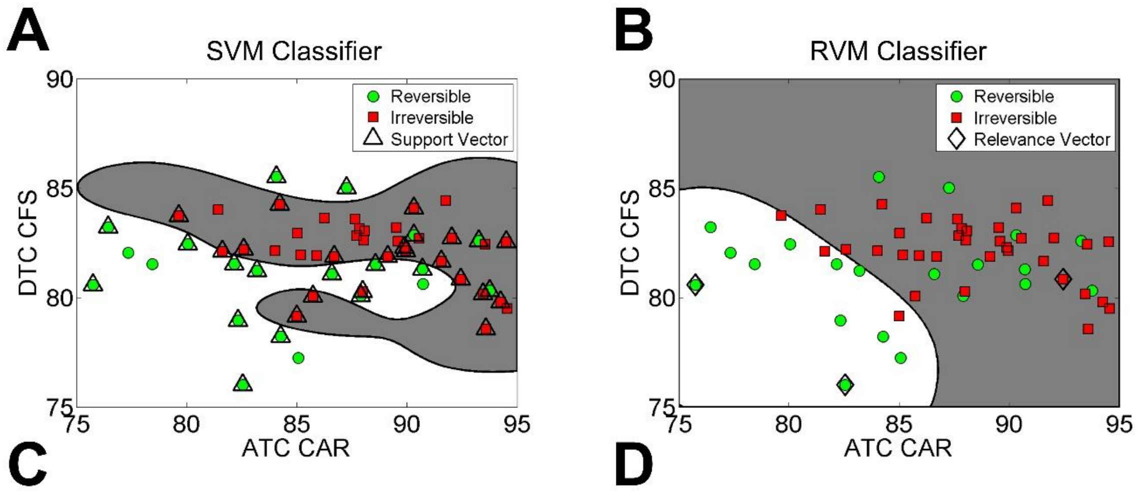

C

Recall	94.44%
Specificity	85.71%
Precision	91.89%
F1 Score	93.15%
Accuracy	91.23%
[σ,C]	[0.8,6]
No. of SV	36 (63.16% of 57)

D

Recall	91.67%
Specificity	52.38%
Precision	76.74%
F1 Score	83.54%
Accuracy	77.19%
s	6
No. of RV	3 (5.26% of 57)

Supp. Fig. 5. Prototype of Kernel Machine predictive models using ATC CAR and DTC CFS. **A).** The distribution of reversible and irreversible patient data in the 2D feature space of ATC CAR and DTC CFS. The SVM was optimized to build the predictive model using cross validation. The solid line between the white and gray domains is the SVM decision boundary between the two groups of patients. There is an overfitting risk as more than half of the samples are support vectors. **B).** The distribution of reversible and irreversible patient data in the 2D feature space of ATC CAR and DTC CFS. The RVM was optimized to prototype the predictive model. The solid line between the white and gray domains is the RVM decision boundary between the two groups. **C).** The performance metrics of the discrete SVM predictive model using ATC CAR and DTC CFS. **D).** The performance metrics of the prototyped RVM predictive model using ATC CAR and DTC CFS.

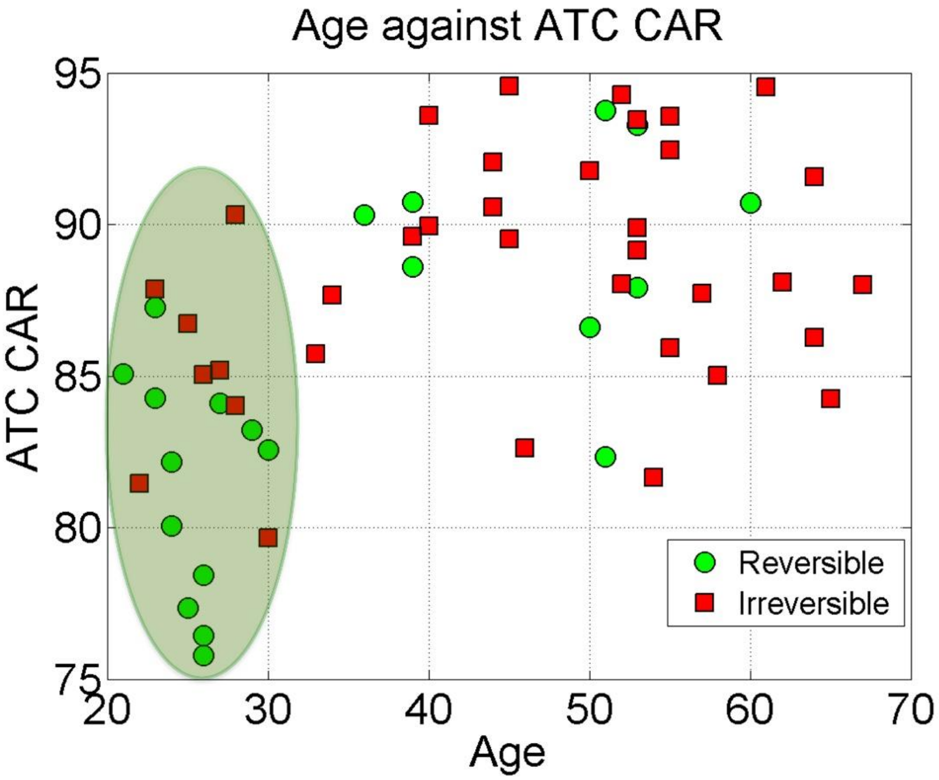

Supp. Fig. 6. The correlation between patient age and HCV-induced liver fibrosis reversibility. Patients <30 years of age have much lower risk of irreversible fibrosis post-SVR treatment as the ATC CAR is much lower than the patients who are >30 years of age.

Supp. Table 1. Nomenclature and explanations of selected key parameters for SHG-TPE quantification.

Abbreviations	Full Name	Explanation
	**INTENSITY**	
CAR	Collagen Area Ratio	Ratio of Collagen area within the Region Of Interest (ROI)
CART	Collagen Area Ratio In Tissue	Ratio of Collagen area within the Tissue Area
CFD	Collagen Fiber Density	SHG intensity density (sum of pixels intensity divided by area of collagen)
UCFD	Unsaturated Collagen Fiber Density	SHG intensity density of unsaturated collagen
TAR	Tissue Area Ratio	Ratio of Tissue area with the ROI
TD	Tissue Density	TPE intensity density
	**STRUCTURE**	
CFL	Collagen Fiber Length	The length of a given collagen fiber
CFT	Collagen Fiber Thickness	The average thickness of collagen fibers.
CRI	Collagen Reticulation Index	Ratio of the number of branch points to the skeleton area
CARD	Collagen Area Reticulation Density	Ratio of the number of branch points to the collagen area
TARD	Tissue Area Reticulation Density	Ratio of the number of branch points to the tissue area
CnFBpR	Collagen Fiber Branch points Ratio	Ratio of the number of branch points to the collagen fibers
	**TEXTURE**	
ContrastCn	Contrast property of the Gray-Level Co-occurrence Matrix (GLCM) of the SHG channel	Returns a measure of the intensity contrast between an SHG pixel and its neighbor over the whole image
CorrelationCn	Correlation property of the Gray-Level Co-occurrence Matrix (GLCM) of the SHG channel	Returns a measure of how correlated an SHG pixel is to its neighbor over the whole image.
HomogeneityCn	Homogeneity property of the Gray-Level Co-occurrence Matrix (GLCM) of the SHG channel	Returns a value that measures the closeness of the distribution of elements in the SHG GLCM to the GLCM diagonal.
EnergyCn	Energy property of the Gray-Level Co-occurrence Matrix (GLCM) of the SHG channel	Returns the sum of squared elements in the SHG GLCM. Also called uniformity.
ContrastTissue (similar to contrastCn but for the TPE channel)	Contrast property of the Gray-Level Co-occurrence Matrix (GLCM) of the TPE channel	Returns a measure of the intensity contrast between a TPE pixel and its neighbour over the whole image

Supp. Table 2. The frequency of fibrosis reversal in patients treated with different direct acting antivirals.

Treatment	Reversible (N=21)	Irreversible (N=36)	Total
	No. of Patients		
SOF	7	14	21
PR	1	2	3
DCV	6	8	14
TMC	6	7	13
Grazoprevir	1	5	6
Total	21	36	57

Abbreviations: DCV: Daclatasvir; PR: pegylated-interferon and ribavirin; SOF: Sofosbuvir; TMC: Simeprevir
